# Deep and ongoing response of castrate-resistant prostate cancer on very low-dose enzalutamide in an elderly chemotherapy–naïve patient: a case report

**DOI:** 10.1007/s00280-021-04269-3

**Published:** 2021-04-04

**Authors:** Emmy Boerrigter, Thomas Havenith, Nielka P. van Erp, John-John B. Schnog

**Affiliations:** 1 Department of Pharmacy, Radboud University Medical Center, Nijmegen, The Netherlands; 2 Department of Clinical Pharmacy, Curaçao Medical Center, Willemstad, Curaçao; 3 Department of Hematology-Medical Oncology, Curaçao Medical Center, Willemstad, Curaçao

**Keywords:** Enzalutamide, Exposure, Pharmacokinetics, Castration-resistant Prostate cancer

## Abstract

**Background:**

Enzalutamide is an orally administered drug that blocks signaling in the androgen receptor with clinical activity in both chemotherapy–naive and post-chemotherapy patients with castrate-resistant prostate cancer (CRPC). Enzalutamide is generally well-tolerated, but dose reductions are nonetheless needed in case of side effects.

**Case:**

An 82-year-old patient with chemotherapy–naive metastatic castration-resistant prostate cancer was treated with a very low dose of 40 mg enzalutamide once daily. The trough levels of enzalutamide and the active metabolite N-desmethylenzalutamide were 4.5 mg/L and 3.0 mg/L, respectively. This exposure provided a long-term response without any significant side effects.

**Conclusion:**

Low doses of enzalutamide may be efficacious, while also reducing the risk of side effects. Furthermore, employing a lower dose would reduce healthcare costs and increase access to enzalutamide. Studies exploring the efficacy of lower enzalutamide doses are warranted.

## Introduction

Enzalutamide is an orally administered drug that blocks signaling in the androgen receptor with clinical activity in both chemotherapy–naive and post-chemotherapy patients with castrate-resistant prostate cancer (CRPC) [1, 2]. The maximum-tolerated dose (MTD) in a phase 1–2 study was 240 mg once daily (qd) and the most common side effect was dose-dependent fatigue, which generally resolved following dose reduction [3]. The dose studied in phase 3 trials and approved by the Food and Drug Administration (FDA) and European Medicines Agency (EMA) is 160 mg qd [1, 2]. However, on this dose still approximately 36% of the patients reported fatigue in the PREVAIL trial. Furthermore, the post-hoc analysis revealed a higher rate of falls in elderly patients (> 75 years) treated with enzalutamide as compared to placebo (19.2% vs. 7.9%) [4]. Also, a sub-group analysis in the AFFIRM study demonstrated an increased rate of fatigue (39.7 vs 31.6%) for men older than 75 years. In addition, enzalutamide is associated with depression and has a negative effect on cognitive function in especially elderly patients [5, 6]. The mechanism for these side effects is not yet fully understood, but it was shown in rodent studies that enzalutamide and its active metabolite N-desmethylenzalutamide (NDME) penetrate the central nerve system (CNS) [7]. The hypothesis is that the direct exposure of enzalutamide and its active metabolite NDME in the CNS cause side effects such as fatigue and cognition impairment.

No exposure–response relation was observed for enzalutamide and its active metabolite NDME [8, 9]. In the phase 1 trial of enzalutamide, [18F]-fluorodihydrotestosterone positron emission tomography (FDHT PET) scans revealed that enzalutamide substantially displaced FDHT binding with a maximum effect seen at 150 mg (corresponding with a C_trough_ of 11.4 mg/L) that was only minimally higher than the effect observed at 60 mg (corresponding with a C_trough_ of 5.0 mg/L)[3]. This might suggest that androgen receptor binding could already be saturated at serum levels of 5.0 mg/L enzalutamide. Therefore, a minimum trough concentration of 5.0 mg/L could be considered as a target for exposure, which can be achieved at doses of 60 mg qd enzalutamide. We describe a case of a patient successfully treated with 40 mg qd of enzalutamide.

## Case presentation

In 2018, an 82-year-old Afro-Caribbean male was referred from urology to medical oncology with a rising prostate-specific antigen (PSA) serum level while under treatment with goserelin 10.8 mg implanted subcutaneously every 12 weeks. He underwent a trans-urethral prostatectomy for a Gleason 7 (3 + 4) adenocarcinoma of the prostate in 2011. With PSA progression shortly thereafter, he started treatment with goserelin and had castration level serum testosteron at PSA progression in 2018. At that time his serum PSA was 33.9 ng/mL, he had new lower backpain and a CT scan revealed one sclerotic metastasis in the 2nd lumbar vertebra (L2). He received palliative radiotherapy on L1–3 (5 × 4 Gy). Besides prostate cancer the patient also had hypertension which was controlled with candesartan 8 mg qd only. Surprisingly for an 82-year-old patient no other comedication was used. He was not on any form of alternative medicine. His World Health Organization (WHO) performance status was 1.

Even though his pain resolved in the ensuing weeks his serum PSA increased to 74.9 ng/mL. Liver and kidney function were normal. He was prescribed enzalutamide 160 mg qd. After eight weeks his PSA declined to 7.83 ng/mL. Our patient revealed he had only been taking one 40 mg capsule a day from start of treatment, because he thought one pill would be ‘enough’. Persuading him to take a higher dose was unsuccessful, since he was responding using only 40 mg qd. He tolerated enzalutamide well and suffered no significant side effects. Four months after starting, his PSA further declined to 0.46 ng/mL with a PSA nadir of 0.05 ng/mL 8 months after initiating enzalutamide treatment. After a total treatment period of one year he decided to take a ‘drug holiday’ as he was feeling fine and ‘wishes as little medication as possible’. Six months later his PSA was rapidly rising after which he started with enzalutamide 40 mg qd again. His PSA declined from 17.4 ng/mL to 0.9 ng/mL within 3 months after restart on enzalutamide. We determined the enzalutamide trough level (drawn 23 h after the previous dose) with a validated method as previously described [10]. The trough levels of enzalutamide and the active metabolite NDME were 4.5 mg/L and 3.0 mg/L, respectively. At the time of writing, our patient has been on enzalutamide for another 9 months with a sustained PSA response until the last measurement when his serum PSA increased from 2.5 mg/L (at 7 months) to 8.2 mg/L (at 9 months) which is defined as PSA progression according to the Prostate Cancer Working Group Criteria 3 (PCWG3).

## Discussion

This case report shows long-term effectiveness of a low dose enzalutamide (40 mg qd) in an 82-year-old patient with metastatic CRPC. No significant side effects were observed during the treatment period. In the PREVAIL trial the median time until PSA progression was 11.2 months and the median time that patients received enzalutamide was 16.6 months. Our patient has sustained response during approximately 21 months (12 months during the first period and 9 months during the second period). This is not the first case describing a good response to low dose enzalutamide. Natchagande et al. reported another case of an 87-year-old patient treated with 40 mg enzalutamide who showed a good response without relevant toxicity[11]. Furthermore, a recent Caribbean retrospective chart review suggested that an enzalutamide dose ≤ 80 mg qd (*n* = 16) leads to comparable responses in elderly patients with metastatic prostate cancer as compared to patients on 160 mg qd (*n* = 43)[12]. In neither studies plasma trough levels of enzalutamide and its metabolite NDME were measured.

Curaçao is an island in the Caribbean with the main inhabitants being of African descent. The incidence of prostate cancer and prostate cancer mortality in the people of African descent is high compared to other ethnicities[13]. This population is generally underrepresented in registration trials, hence translation of trial findings to this specific population is difficult. Efficacy and pharmacokinetic data presented in this case report, therefore, adds to the available knowledge.

Our patient was exposed to enzalutamide in a concentration of 4.5 mg/L and the active metabolite NDME was 3.0 mg/L. Enzalutamide is rapidly absorbed and the bioavailability of enzalutamide is estimated to be at least 84% [14]. Food does not have a clinically significant effect on absorption [14]. Enzalutamide is hepatically metabolized, primarily by Cytochrome P4502C8 (CYP2C8) with minor CYP3A4/5 involvement. CYP2C8 is primarily responsible for metabolizing enzalutamide into NDME [14]. CYP2C8*2 is the most prevalent (11.5–18.3%) variant in people of African descent, which can lead to a poor metabolic phenotype [15]. The elimination half-life of enzalutamide is 5.8 (2.8–10.2) days [14]. At steady state the enzalutamide:NDME plasma ratio is approximately one. In our patient, the enzalutamide concentration was 50% higher than the NDME concentration. This deviation in ratio might be caused by a CYP2C8 polymorphism. In addition, a CYP3A4 polymorphism (e.g. *22) leading to reduced CYP3A4 metabolism, cannot be excluded. In a pharmacokinetic interaction study of enzalutamide coadministration of itraconazole (a strong CYP3A4 inhibitor) increased the area under the plasma concentration–time curve from zero to infinity (AUC_0-∞_) of enzalutamide by 1.4-fold and NDME by 1.2-fold [16]. Therefore, an important role for potential CYP3A4 polymorphisms is not expected. As detection of these polymorphisms would not have impacted treatment of our patient their potential presence was not assessed.

The pharmacokinetics of enzalutamide is dose proportional over a dosage range from 30 to 360 mg/day [8]. The average trough concentrations of enzalutamide and NDME are 11.4 and 13.0 mg/L at 160 mg qd, respectively [14]. In this context, the measured enzalutamide level in this case is higher than expected while the NDME level is in line. Potentially due to polymorphisms as explained before.

The within patient variability of enzalutamide and NDME is very low (8.8% and 7.3% respectively) [17]. In our patient, blood was drawn at steady state and a drug–drug interaction was excluded. Based on the pharmacy prescription data, the patient seemed to be adherent to his medication. Therefore, we assume that the measured exposure is reliable.

The enzalutamide exposure was only slightly below the level at which androgen receptor binding may be saturated (5 mg/mL enzalutamide), hence explaining the good, sustained response to therapy. However, the mutational status of our patient was unknown. Since our patients was chemotherapy–naïve, the incidence of androgen receptor alterations (e.g. amplifications, structural variants and mutations) is expected to be low. Possibly, for patients with androgen receptor alterations a higher exposure as target for effective treatment is necessary. More research is necessary to confirm this hypothesis.

In anticancer drug development, the dose entering phase 3 clinical trials is often close to the MTD. This may be a higher dose than needed for attaining meaningful clinical responses. This does not only increase risk of side effects but also costs to society. As recently proposed, the concept of interventional pharmacoeconomics is aimed at decreasing prescribing costs by developing new dosing regimens while maintaining equivalent treatment efficacy [18]. A recent example of such an intervention was with abiraterone (non-fasting 25% of dose demonstrating non inferior PSA decrease to 100% fasting dose) [19]. The repeated deep PSA response in our patient suggests that, at least in patients who are chemotherapy–naive, low doses of enzalutamide may not only be effective and significantly reduce costs but also increase access in resource limited countries. Studies targeting lower enzalutamide doses are therefore warranted.

Our 82-year-old patient experienced no CNS side effects, such as fatigue or cognition and memory disturbance. Possibly due to the low exposure, these side effects may not have occurred. Although there is evidence that the CNS side effects of enzalutamide are dose-related due to high systemic exposure, up until now the relation between the exposure and the rate of fatigue has not been proven [9, 17]. Future research should focus on investigating whether lower doses of enzalutamide with lower exposure can prevent CNS side effects (especially in frail elderly patients). Currently, we are running a prospective trial to study this hypothesis (Clinicaltrials.gov NCT03927391).

## Conclusion

A very low dose of enzalutamide (40 mg qd) provided a long-term response without any significant side effects in an 82-year-old patient with chemotherapy–naive metastatic CRPC. Further research is needed to investigate whether low doses of enzalutamide can reduce side effects while preserving efficacy.
